# Perceptions of nursing students regarding responsible use of social media in the Eastern Cape

**DOI:** 10.4102/curationis.v38i2.1496

**Published:** 2015-07-24

**Authors:** Thando Nyangeni, Suzette du Rand, Dalena van Rooyen

**Affiliations:** 1 Department of Nursing Science, Nelson Mandela Metropolitan University, South Africa; 2 School of Clinical Care Sciences, Nelson Mandela Metropolitan University, South Africa

## Abstract

**Background:**

Social media have become a popular communication system that has transformed communication from the traditional to the Web-based model. Because social media use has no limitations to place and time, it is now used extensively at clinical facilities. Social media use is becoming a popular activity amongst students at Nursing Education Institutions (NEI) in South Africa. However, lack of accountability and unethical use of social media by nursing students in South Africa has been reported.

**Objectives:**

The aim of the study was to explore and describe the perceptions of nursing students regarding responsible use of social media.

**Methods:**

A qualitative, descriptive, explorative and contextual research design was used to explore and describe the perceptions of nursing students regarding the responsible use of social media. Twelve nursing students registered for the undergraduate nursing degree were purposely selected and interviewed individually using a semi-structured interview method.

**Results:**

The results of this research study demonstrate that nursing students use social media irresponsibly. Nursing students experience blurred boundaries between personal and professional lines and lack accountability when using social media.

**Conclusion:**

The extensive use of social media in the clinical environment, by healthcare students, requires a joint effort by Nursing Education Institutions and healthcare facilities to ensure that social media are used in an ethically acceptable manner. The implementation of the recommendations of this research study could positively influence legally and ethically acceptable use of social media at healthcare facilities.

## Introduction

The phrase ‘social media’ refers to a set of online tools that are designed for and centred around social interaction. According to Bertot, Jaeger and Hansen (2012:30) the intention of these online tools is to enable users to interact with one another and communicate, edit and share content. The content of social media is, therefore, user-generated, and allows large groups of geographically dispersed people to produce valuable resources of information and to access rare expertise (Bertot *et al.* 2012:30). Boyd and Ellison define social networking sites as:

Web based services that allow users to construct a public or semi-public profile within a bounded system, articulate a list of other users with whom they share a connection, and view their list of connections and those made by others within the system. (2008:211)

Social media include a mixture of Web-based technologies and services such as blogs, microblogs (e.g. Twitter), social video sharing services (e.g. YouTube), text messaging (e.g. Wikis), virtual worlds (e.g. Second Life) and social networking services (e.g. Facebook, MySpace). Information and communication technology, especially via the Internet, continues to play a significant role in shaping current and future social, economic and personal structures (Akoh 2012:3). According to Newbold and Campos (2011:6) healthcare facilities are taking advantage of the reach provided by social media to publish health information and to engage with their customers. Social media now seem to have replaced face to face contact as a means of communication between people (Crawford 2009:532). The Mayo clinic, for example, is one such pioneering healthcare facility that has used social media to allow patients to interact with one another and with health professionals to seek health information (Newbold & Campos 2011:6).

Social media have grown rapidly and have become a social communication tool of choice, especially for young professionals and students, because it is cost-effective and convenient to use (Cain & Fink 2010:1). The convenience of social media allows users to communicate with other people without any limitations of time, place and distance (Schmitt and Lilly 2012:181). The growth, of social media as emerging Internet communication tools, is expected to add value and benefit to existing methods of distance education (Akoh 2012:6). For students in tertiary education institutions, the convenience of social media as communication tools is an advantage because the student does not have to attend class, but can interact with the class colleagues and lecturers from home or at work. Social media can facilitate online learning, allowing flexibility that accommodates adult learning through convenience of time and place (Akoh 2012:9). Social media are viewed as enabling technologies that support blended learning solutions, and can encourage active learning and knowledge construction through peer-to-peer interaction (Holden & Westfall 2010:14). Another purpose that social media can serve is enhancing clinical expertise in healthcare. Expert guidance for learners and facilitators can be sought from specialists throughout the world; this is particularly beneficial for nurses who work in under-resourced areas, because they can send messages or even pictures to people in resourced areas in order to receive the best advice to deliver better patient care (Martinez-Garcia *et al.* 2013:978).

Whilst social media offer benefits for students and professionals, there are legal and ethical risks associated with their use. The improper and irresponsible use of social media carry significant risks for users, employers and organisations, such as tertiary education institutions and healthcare facilities (National Council of State Boards of Nursing 2013:14). Inappropriate use of social media can potentially damage personal integrity, nurse-patient relationships, nurse-colleague relationships, and current and future employment opportunities (New Zealand Nurses Organisation 2012:2). Privacy and online security risks pose new challenges which institutions, academic bodies and teachers should consider when they develop policies for social media use (Akoh 2012:18). Of late, there have been instances of inappropriate use of social media involving nursing students and other healthcare personnel in America (Chandra & Chatterjee 2011:453).

Despite the availability of information regarding the use of social media by nurses and nursing students in other countries, there is a paucity of research regarding the use of social media by nursing students in South Africa. The goal of this research project is, therefore, to investigate the perceptions of nursing students regarding the responsible use of social media.

## Problem statement

As a lecturer at a South African Nursing Education Institution (NEI), the researcher witnessed and had been informed by his colleagues, in this and other NEIs, that misconduct might be taking place regarding social media usage. The researcher also became aware of the indiscriminate use of social media by nursing students when patient-related information (text and pictures) was sent to him, friends, colleagues and other nurse educators. On investigation, he also became aware of pictures – of patients, babies and other patient-related information – that were circulated between students and nurse educators, by students in clinical practice.

Nurses have a legal and ethical duty to maintain the privacy and confidentiality of their patients. The practice of divulging patient information is unethical and illegal because the students were not following the correct procedures when they took and disseminated photos of patients. The privacy and confidentiality of the patients were, thus, not upheld. When patients become aware that their information is being disseminated on social media, they might be reluctant to give full details of their medical conditions to healthcare personnel and might lose confidence in the healthcare system. It is, thus, crucial for the educators as bastions of professionalism, to guide the nursing students whilst protecting the dignity of the patient population.

The suspected improper use of social media by nursing students prompted the researcher to explore this problem further. The aim of this research study was, therefore, to gain a better understanding of students’ perceptions regarding responsible use of social media.

## Research question

Having identified the problem, the researcher aims to answer the following research questions:

1. What are the perceptions of nursing students regarding the use of social media?
2. How do nursing students use social media in the clinical environment?

### Goal of the study

The goal of the study is to establish the perceptions of nursing students regarding the responsible use of social media.

### Objectives of the study

The objectives of the study are:

- To explore and describe the perceptions of nursing students regarding the use of social media at NEIs and in health facilities.
- To identify the ways in which nursing students use social media in the clinical environment.

## Research methods and design

A qualitative, explorative, descriptive and contextual design was used to achieve the objectives of the research study.

The population for this study was the nursing students who were currently studying in the undergraduate nursing (degree) programme at an NEI. This population was comprised of the majority of the nursing students studying in this NEI and included native South African and international students. This was the appropriate population for the study because the researcher could draw conclusions about the perceptions of nursing students regarding the use of social media in the Eastern Cape NEIs.

Non-purposive sampling was used for this research study. The researcher relied entirely on his judgement, as the chosen sample was composed of the elements that contained the characteristics representative or typical attributes of the population, that best serve the purpose of the study (Strydom 2012). To obtain the sample, the following criteria for inclusion were used:

Students in their first, second, third and fourth year of the undergraduate nursing programme were included because the researcher wanted to understand their perceptions regarding the use of social media, and compare the students who have had clinical experience with those who had not had clinical exposure. However, none of the first year undergraduate nursing degree students volunteered to participant in the study.

## Data collection

Gate keepers were approached by means of a formal letter requesting permission to conduct research at the NEI. The aim of this formal letter was to gain permission to conduct the research study. The researcher requested class lists from the NEI where the research study was conducted and used electronic mail (email) to invite students who were registered in the undergraduate nursing degree programme to participate in the research study. The researcher put his contact details on the email to enable students willing to participate to contact him directly. After gaining ethical approval to conduct the research study, arrangements were made with the students who agreed to participate for the interviews to take place, at a time that was suitable for them, and in a setting that was convenient and comfortable. Students who indicated their willingness to participate in the research study were contacted by the researcher, using a contact method chosen by them.

Semi-structured individual interviews were used to collect data. The use of these interviews allowed the researcher to follow an interview schedule, or guide, with basic questions which provided enough flexibility to probe and explore deeper in order to gain clarity on issues that emerged during the course of the interview (Wagner, Kawulich & Garner 2012:134). The researcher used pre-determined questions which only served to guide rather than dictate the interview schedule. The number of participants interviewed for this research study was determined by data saturation, which is a point at which no new themes emerged from the interviews (Wagner *et al.* 2012:62). The perceptions of nursing students regarding the responsible use of social media were explored. The interviews were recorded electronically with an audio recorder and were transcribed verbatim by the researcher himself.

## Data analysis

Data analysis was performed at the same time as data collection. Coding and data analysis were performed according to the eight steps suggested by Tesch (in Cresswell 2009:186). Coding was undertaken by the researcher and by an independent, experienced researcher and coder who holds a doctoral degree. The interviews were transcribed and coded in order to assess the themes that arose. The independent coder was given printed transcripts of each interview and the voice recordings by the researcher. The independent coder was asked to sign a declaration of confidentiality in order to safeguard the rights of the participants. After the coding of themes, the researcher and the independent coder reached a consensus regarding the final themes and sub-themes.

### Research participants

Twelve students were interviewed, eleven of whom were South Africans and one, an international student. The participants’ ages ranged from 20 to 28 years. Two of the participants were males and the rest (10) were females.

### Establishing trustworthiness

Trustworthiness in qualitative research means methodological soundness and adequacy (Holloway & Wheeler 2002:254). To achieve trustworthiness, the following criteria were applied:

- credibility
- dependability
- confirmability
- transferability (Polit & Beck 2012:584).

## Results

All the participants subscribed to two or more social networking sites for the purpose of communicating and keeping in touch with friends, relatives and colleagues. Facebook seems to be the most popular social networking site used by students, followed by WhatsApp, Twitter, YouTube, LinkedIn, Blackberry Messaging, Mxit, Instagram and Google+. Students mentioned that they use social media for academic purposes both in the classroom and clinical practice environments. All participants used a cellular phone and a personal or university computer to connect to social media. From the interviews two themes emerged:

- There is no awareness of responsible use of social media amongst nursing students.
- There are blurred boundaries between private and public roles and a lack of accountability.

Each theme and related sub-themes will be briefly elaborated on below and discussed in details in the discussion section.

### Theme One: There is no awareness of responsible use of social media amongst nursing students

The first theme that emerged from the interviews is that nursing students have no knowledge or awareness of responsible use of social media and, therefore, use social media irresponsibly. Students admitted that there is no responsible use of social media. Students stated that each of them perceives responsible use of social media differently. One participant went on to state that she is not using social media responsibly:

‘I think there is no responsible use on social media. I've seen and from my own personal experience … because what is responsible to me, may not be responsible to somebody else.’

### Sub-theme One: Contravention of patients’ rights to privacy and confidentiality

All students indicated that they took pictures, recorded video and audio clips of patients and of clinical interactions involving patients and posted this information on social media. Students shared that they used social media to send this information to friends and relatives in order to inform them about what they experience in healthcare facilities and to entertain them:

‘We want to tell them what we see and they will also share what they have there. Some are not nursing friends, we just want to overwhelm them and we want them to laugh about this or we want to scare them and just show them what we get to see in our work place.’

### Sub-theme Two: Patient's medical conditions were laid bare by students on social media

Students shared that they posted everything they could about their patients. Students stated that they shared detailed information about patients’ medical conditions, including pictures of affected body organs, such as patients’ genitals, with friends, families and colleagues. Students shared that they took pictures of patient's genitals in order to teach the public about sexually transmitted infections. Students went on to disclose that the patient's pictures were taken from clinical facilities:

‘ … Just the genitals or maybe mention it that it was in a clinic or it was in a hospital.’

### Sub-theme Three: Failure to obtain informed consent

Students do not obtain informed consent before taking and distributing a patient's photos, audio and video clips on social media. In some cases students obtained the consent before taking photos, videos or audio clips of patients but did not obtain consent for sending information to third parties:

‘We won’t inform the patient, we will just steal the picture and circulate it. Some patients we ask permission and [*they*] will give us [*the consent*] willingly because maybe they want us to learn about the situation or thing.’

It is clear that nursing students understand the obligation to obtain consent before taking and distributing patient information on social media, but they seem to justify themselves for not complying with the obligation to obtain informed consent. One of the students stated that attempting to obtain consent from the hospital authorities was a long process which could potentially jeopardise his chances of taking the photos before the patient was discharged home:

‘You just wanna take the short cut it's because you know sometimes you just need those things now for your own … You don’t wanna go up there and ask you know moss to follow those criteria, it's a long process.’

### Sub-theme Four: Exploitation and manipulation of vulnerable patients and lack of advocacy

Students shared that they inappropriately took advantage of the vulnerability of the patients by taking pictures of them under the pretext that they were doing it for educational purposes. Students shared that they established a relationship of trust with the patients, and when they felt that the patient was comfortable with them, they took the pictures and distributed them to other people:

‘You can first like see a patient for like a couple of days, be friendly and then days later you can ask …’

Some students felt that their colleagues failed to play the role of being an advocate for the patients. It appears that students took advantage of the vulnerability of their patients and went on to take pictures of patients and posted them on social media. Patients, who could not exercise choices, such as babies and the mentally ill, were victims of the students’ irresponsible social networking behaviour. The need to inform other people about their patients’ conditions seems to be more important for students than to advocate for their patients:

‘Those babies with congenital defects, people with extensive side effects like in psychiatry when they do those hallucinations and they don't really know where they are. And also if maybe you are cleaning patient's wound or how it is then you take a picture and send …’

### Sub-theme Five: Clinical interactions and medical procedures are recorded and posted on social media

The information students shared on social media is not limited to that of individual patients. Students took and disseminated video or audio clips of clinical interactions to their friends. One of the participants shared that she received a video recording of a psychotherapy session and pictures of patients with head injuries and pictures of surgical procedures performed in the operating theatre:

‘I have received a video of a, it was a session with a psychologist, in fact, it was not a video, it was a recording. The patient is having psychological counselling session and then others were pictures of wounds and others were pictures of patients with head injuries and others were pic[*ture*]s from theatre of the operations that were being done there … Just videos of demonstrations by other nurses and doctors in the hospital.’

### Sub-theme Six: Competition to receive recognition for being the first person to post information on social media

Students felt that posting ‘special photos’ online is somewhat competitive and is associated with rewards. Students shared that they could not miss the opportunity to capture ‘that special’ moment on camera and post it online. They often felt a sense of satisfaction and triumph when they were the first to see a patient who had a certain condition about which they were taught in class. Being the first person to see a medical condition seems to prompt students to post even more information about specific patients online and this makes students seem resourceful to those students who had not seen a patient with a specific medical condition in the clinical facilities:

‘It's something that I will type out … okay this is what I saw, this is what the patient presented with. Remember this lecture said that you will see this this and that? And then I will type it and then they’ll be kind of probing you with questions … when I get back to res[*idence*] and something that I will kind of brag about in that group.’

### Sub-theme Seven: Awareness of the digital footprint and misrepresentation of the nursing profession, the Nursing Education Institutions and healthcare facilities

Students shared that they are aware that information that is disseminated on social media often goes beyond the intended recipients. Some students demonstrated awareness that when information is shared with other people on social media, it remains in circulation long after it has been deleted from one's social networking site. Students also seem to know that the kind of information that they post on social media could damage the reputation of the nursing profession, the NEI and the healthcare institutions:

‘Internet doesn't or it's not gonna just end. Ten years from now we're all gonna have kids then they are gonna be finding all that what we are doing now and it's coming back to us.’

Although some of the students seemed to be aware of the fact that information posted on social media can be traced back to the original author, some of them unaccountably believe that if they post the information on social media and thereafter remove it, it will be difficult to recover. One student believes that if he deletes the information he posted on social media, no one can trace that message back to him. Another student further stated that if an investigation was launched into his posts on social media, it would be a very long and possibly futile exercise to do so:

‘I delete it ‘cause I work in that ward so nobody … if somebody touches my phone nobody can see the pic because it's out of my phone. If it's not there in my phone even if it's within the circulation.’

### Theme Two: Blurred boundaries between private and public roles and lack of accountability

Students shared that they experience difficulty in separating their professional and personal lives when using social media. All participants whom the researcher interviewed stated that their private and professional lives are inseparable. They said that posting on social media, as nursing students, should not be any different from posting as a private citizen:

‘It's very difficult to separate myself from my career … So it's difficult to be 2 people.’

### Sub-theme One: Students establish inappropriate relationships with their patients and post about those relationships on social media

It is clear that students did not keep their relationships with patients of professional nature, but went on to establish casual relationships with patients. One participant indicated that her colleagues established casual, unprofessional relationships with patients and took photos with these patients and posted them on social media:

‘When we were doing psychiatry in the places that we visited … most of the time [*students*] create relationships with patients and then maybe, maybe in the last days then take pictures … I've seen, I've seen some of the classmates, with pictures of patients.’

### Sub-theme Two: Justification for inappropriate use of social media

Students provided reasons to justify their inappropriate social networking behaviour. Students shared that they carry their cellular phones on them whilst at clinical facilities and use them to take pictures which are eventually posted on social media. Students shared that it is risky to leave their cellular phones in hospital lockers because their safety is not guaranteed. Students also blamed staff working at clinical facilities who often use social media whilst providing patient care:

‘The places that we are allocated to they don't have lockers for the many students that are placed in that particular ward … All your prized possessions must be with you because you don't know what's gonna happen.’

## Ethical considerations

All research studies must comply with sound ethical practices and standards, and there must be a balance between contributing to science and protecting the rights and dignity of human subjects (Pera and van Tonder 2012:326). Research ethics provide guidelines for responsible conduct. The researcher undertakes to consider and adhere to all ethical principles at every stage of the research process, to safeguard the integrity of the research study, and to protect the research participants. To achieve trustworthiness in this study, the following ethical principles were adhered to:

- beneficence and non-maleficence
- autonomy
- justice
- privacy and confidentiality.

### Entry to site

Researchers must gain entry into sites that are suitable for the enquiry and must seek the approval of the gatekeepers (Polit & Beck 2012:184). The researcher supplied adequate information to help the gatekeepers understand the benefits of the research. This information must cover the following:

- the purpose of the research and identify who the beneficiaries would be
- why the site was chosen
- how much disruption could be expected
- the resource requirements
- the maintenance of ethical guidelines
- explain how results would be reported (Polit & Beck 2012:184).

The gatekeepers in this study were the Head of the Nursing Science Department, the Dean in the Faculty of Health Sciences and the Deputy Vice Chancellor: Research and Engagement. Formal letters requesting permission to interview the participants were written to the gate keepers. The researcher submitted the research proposal to the Health Sciences Faculty and the Department of Nursing Science research committees for approval and gained permission to undertake the research before beginning the fieldwork.

### Gaining ethical permission to do the study

The research proposal was sent to the research committees in the Department of Nursing Science and the institutional Research Ethics Committee of the NEI for approval. The researcher also sought the approval of the REC-H committee because students would be interviewed in this study. Further, the researcher followed the prescribed ethical principles, throughout this research study, namely:

- protection from harm and exploitation
- beneficence
- respect for human dignity
- autonomy
- justice
- privacy and confidentiality.

## Discussion

This research study shows that nursing students enjoy posting patient-related information on social media. Students acknowledged that they are not using social media responsibly. Students shared that they are not aware of any universally acceptable description of responsible use of social media. As a result students felt that this lack of a universally acceptable description of responsible use of social media leads them to have different perceptions of the responsible use of social media.

A number of articles about social media are emphasising the need for development of guidelines for responsible use of social media. Ventola (2014:498) encourages organisations to establish guidelines for appropriate use of social media in order to prevent legal and ethical violations. Policies for social media use should address issues like discrimination, harassment, productivity, use of cellular phones, damage to the organisation's reputation, amongst other issues (Ventola 2014:498). Baker (2013:505) states that there are no guidelines on how to deal with social networking as a first step to establishing policies for social networking.

The information about patients that students post on social media has large amounts of detail that could potentially expose the identity of the patients. The patients’ rights to privacy and confidentiality were, therefore, infringed by students. Chretien *et al.* (2009:1312) found that students posted information describing clinical experiences with enough detail that patients could potentially be identified. It is common for nurses to share information on social networks to impress other people and to share information about their professional experiences, but this must not take place at the expense of the patients’ right to privacy and confidentiality (Klich-Heartt & Prion 2010:58). In a study on teenage patients regarding privacy and social media, it was found that teenage patients used privacy settings in order to protect their medical information from being accessible to other people (Van Velden & El Emam 2013:20). The decision to protect their medical information from being in the public domain on social media shows how important privacy and confidentiality are to patients.

Students shared that the information they post online include pictures of patients’ genitals which they claim are posted to teach the public about sexually transmitted infections. Posting pictures of a patient's genitals is a direct infringement of the patient's right to dignity. A study exploring the administrative experience with online misconduct found that 60% of allopathic schools in the United States of America (USA) reported incidents of students posting unprofessional content online, including details that violate patient confidentiality, use of discriminatory language and behaviour, sexually explicit material and public displays of intoxication (Go, Klaassen & Chamberlain 2012:296). Whilst the use of social media is a tool for dissemination of health information, such a practice is not acceptable if it compromises the rights of other people to dignity. The teaching method students are proposing can, therefore, not be advocated for.

Students stated that they did not always obtain consent from the relevant stake holders before taking and posting patient information on social media. They also shared that they secretly took pictures of patients and distributed them if they felt that they were likely to experience opposition. Palacios-Gonzalez (2014:1) reports that a doctor was fired from a hospital for publishing information on her Facebook page, involving photographs of patients before a surgical procedure. The doctor did not have the patient's informed consent before taking the photographs and posting them online. Chretien *et al.* (2010:70) found that students who were advised to be cautious when using social media felt unfairly constrained. Nurses have an obligation in terms of the law to obtain informed consent from the patients before undertaking any clinical investigation, intervention or research (Stellenberg 2013). In order for the patient to give informed consent, the patient must be empowered with full, accurate and honest information regarding the act for which consent is necessary. Berglund (2007:89) states that giving full information also enables the patient to exercise autonomy in decision making.

Students shared that they befriended their patients in order to make them feel at ease before they took and distributed their pictures. Devakumar *et al.* (2013:4) found that getting consent was perceived to be important when a photo of a child is taken, and the request for consent must be accompanied by sufficient details stating the intended purpose of the photograph. Vasuthevan and Geyer (2013) state that this kind of behaviour amounts to exploitation of patients and a gross violation of human rights, as stated in the Bill of Rights.

Students shared that their colleagues did not play the advocacy role as expected of them. The information they post on social media includes video clips of patients with hallucinations and pictures of babies with congenital abnormalities. ‘Advocacy’ is described by Gilkey and Earp (2009:120) as efforts to support patients and their interests in healthcare. Nurses have a privileged relationship of trust with patients, which is based on the premise that the nurse will always act in the best interests of a patient (Vasuthevan & Geyer 2013). Henry and Molnar (2013:1428) found that second year dental students who were placed in clinical practice for the first time were more likely to engage in unacceptable social networking behaviour, such as taking pictures of patients sitting on dental chairs.

It is clear that students did not only take and distribute pictures, video and audio clips of patients, but recorded and posted video clips of clinical interactions such as psychotherapy sessions and surgical operations in operating theatres. Frankish, Ryan and Harris (2012:182) found that if doctors divulged details of their patients’ confidential medical information, patients would be less likely to reveal important information during a medical consultation. Posting of inappropriate and unprofessional content on social media can damage the reputation of the healthcare workers because potential employers can conduct background checks on the person on social media, and might discover these inappropriate or unprofessional postings (Pillow *et al.* 2013:28).

The posting of patient information on social media was perceived as competitive, motivating and bringing about a sense of accomplishment for nursing students. Research attributes this kind of behaviour to a lack of policies about acceptable social networking. Kind *et al.* (2010:3) found that medical schools that have social media policies guide students in understanding what is acceptable and appropriate social networking behaviour.

Students expressed an awareness of the digital footprint and the fact that they were misrepresenting the healthcare facilities and the NEIs:

‘A digital footprint’ is a trail left by an entity's interactions in a digital environment; including their usage of a mobile phone, the Internet and World Wide Web, mobile web and other devices and sensors. (Eke 2012:1)

Students unaccountably believed that they are smart enough to avoid being identified to have posted patient-related information on social media. The information one shares online can be viewed by other people and once the information is online, it remains there forever (Thatcher 2014:1769). Everything one posts online combines and makes one's digital footprint, which can be traced back to the person that posted the information (Ventola 2014:498). Students shared that they used social media to voice their concerns regarding non-availability of resources, such as linen savers at state hospitals. This could potentially jeopardise the relationship between NEIs and clinical facilities in which nursing students are placed for clinical practice.

Students shared that they experienced blurred lines between public and private roles when using social media because they could not differentiate between public and private personas online. Landman *et al.* (2010:383) found that the dual use of social media, for professional and for personal purposes, causes blurring of lines between one's role as a health professional and one's personal or social role. Cain, Scott and Akers (2009:4) found that students do not have a full understanding of what constitutes private versus public information and the consequences of making private information public. The Nursing and Midwifery Board of Ireland (2013:9) advises nurses to keep a separate personal and professional life online. The more health professionals in training separate their private and professional lives on social media, the less their professional lives will be misinterpreted (Farnan *et al.* 2013:624).

Students made it clear that their colleagues who were placed at mental health facilities established romantic relationships with their patients and took pictures of themselves and these patients and posted them on social media. Chretien *et al.* (2009:1311) also found in their research that medical students posted sexually provocative photographs and sexually suggestive comments requesting inappropriate friendships with patients on Facebook. Students are expected to conduct themselves with honour and integrity. Establishing casual relationships with patients is unprofessional and is against the values of the nursing profession.

Maintaining professional boundaries is important in nursing practice as these boundaries allow for a safe personal connection between the healthcare providers and the patients and clients. Gutheil and Simon (in Baca 2011:196) define a boundary as ‘the edge of appropriate or professional behaviour, transgression of which involves the therapist stepping out of the clinical role’. They add the following:

not all boundary crossings are boundary violations … A boundary crossing is a deviation from classical therapeutic activity that is harmless, non-exploitive, and possibly supportive of the therapy itself … A boundary violation is harmful or potentially harmful to the patient and therapy alike because it constitutes exploitation of the patient, using the therapist-patient relationship as its vehicle. (p. 196)

The researcher found that students cannot differentiate between private and professional roles when using social media. According to Von Muhlen and Ohno-Machado (2013:777), the posting of unprofessional information and breaches of patient confidentiality using social media, especially by healthcare workers, is common, and this behaviour sends the wrong message to students who could adopt inappropriate social networking behaviours as acceptable. Thompson *et al.* (2008:956) found that medical students do not associate negative professional consequences, of their social networking, with their current and future practice of sharing information that could be misinterpreted.

## Recommendations

In the light of the findings and the limitations highlighted above, the following recommendations are suggested by the researcher:

### Recommendations for nursing practice

The following recommendations are proposed for nursing practice:

- The dissemination of the findings of this research study to the public and private healthcare organisations for them to develop policies for social media usage amongst nursing students allocated to their healthcare institutions.
- The development of social networking policies to enforce ethical and legal social networking in the healthcare facilities.

### Recommendations for nursing education

The following recommendations are proposed for nursing education:

- Nurse educators should cascade the information on ethical and legal obligations of nursing students regarding the use of social media by incorporating the topic into their lecturers about ethics and professional conduct.
- NEIs should develop their own policies for social networking that fit the philosophy and the culture of their organisation.
- Disciplinary procedures should also be developed in terms of such policies and upheld, to ensure that all employees and students comply with the policies.
- NEIs should inform their staff by giving them in-service training regarding the appropriate use of social media as social media was not used during their training.
- The expectation of the role models in these organisations should be clearly stated.

### Recommendations for nursing research

The following recommendations are made for nursing research:

- A research study will be useful to determine the effectiveness and the impact of implementation of the guidelines.
- Further research is required to establish the role of the lecturers and hospital personnel in the use of social networking conduct of the nursing students.

### Limitations of the study

It is recognised as with all qualitative studies that the limited sample size and the specific nature of the sample and research context may prohibit generalisation. However, the richness of the data illuminated and explained the experiences of nursing students regarding their use of social media, and enabled the researcher to achieve his goals for this research project. The following are the limitations of this study:

- Data were collected from students in the undergraduate level of the nursing degree programme and, therefore, the experiences of the nursing students in the postgraduate degree and diploma programmes regarding the use of social media are not known.
- The researcher depended solely on the story as told by the participants and had no opportunity to observe the social networking conduct of the nursing students, thus, the researcher made inferences based only on the information that was supplied by the students.

## Conclusion

This research study provided in-depth understanding of the way in which nursing students use social media. Evidence of the irresponsible use of social media by nursing students was identified. Social media is a communication tool for the future, which has been embraced by billions of people throughout the world including at NEIs. The irresponsible use of social media by nursing students in this research study is not and will never be a reason for calling for the banning of the tool at NEIs, but the guidelines have been put forward to guide the nursing profession to implement social responsibly and accountably. The guidelines developed in this research study can be used by the healthcare facilities as a basis for the development of policies for social media use by their staff.
